# 5-Aminolevulinic Acid Multispectral Imaging for the Fluorescence-Guided Resection of Brain Tumors: A Prospective Observational Study

**DOI:** 10.3389/fonc.2020.01069

**Published:** 2020-07-08

**Authors:** Patra Charalampaki, Phileas Johannes Proskynitopoulos, Axel Heimann, Makoto Nakamura

**Affiliations:** ^1^Department of Neurosurgery, Cologne Medical Center, Cologne, Germany; ^2^Witten-Herdecke University, Witten, Germany; ^3^Institute for Neurosurgical Pathophysiology, Medical University Mainz, Mainz, Germany

**Keywords:** brain tumor, 5-ALA = 5-aminolevulinic acid, microscopic surgery, neurological surgery, fluorescence guided surgery

## Abstract

Fluorescence-guided surgery with five-aminolevulinic acid (5-ALA) is the state-of-the-art treatment of high-grade gliomas. However, intraoperative visualization of 5-ALA under blue light remains challenging, especially when blood covers the surgical field and thereby fluorescence. To overcome this problem and combine the brightness of visible light with the information delivered with fluorescence, we implemented multispectral fluorescence (MFL) in a surgical microscope, a technique that is able to project both information in real-time. We prospectively examined 25 patients with brain tumors. One patient was operated on two different lesions in the same setting. The tumors comprised: six glioblastomas, four anaplastic astrocytomas, one anaplastic oligodendroglioma, two meningiomas, 11 metastatic tumors, one acoustic neuroma, and one ependymoma. The MFL technique with a real-time overlay of fluorescence and white light was compared intraoperatively to the classic blue filter. All lesions were clearly visible and highlighted from the surrounding tissue. The pseudocolor we chose was green, representing fluorescence, with the surrounding brain tissue remaining in its original color. When blood was covering the surgical field, orientation was easy to maintain. The MFL technique opens the way for precise and clear visualization of fluorescence in real-time under white light. It can be easily used for the resection of all tumors accumulating 5-ALA. Drawbacks of classic PpIX fluorescence such as hidden fluorescence, intraoperative changes could be overcome with the presence of additional white light in MFL technique.

## Introduction

Malignant brain tumors and especially high-grade gliomas (HGG) are characterized by their aggressiveness and incurability. An influential phase III trial was able to show that mean survival of patients diagnosed with glioblastoma (GBM) is only 14.6 months when treated with radiotherapy and temozolomide (1) and that a mere 9.8% survived for 5 years (2). Apart from the use of radio- and chemotherapy, survival, neurological function, and the patients' quality of life highly depend on the extent and safety of neurosurgical resection (3, 4). For the neurosurgical treatment of HGG, total resection of the tumor while not causing new neurological deficits is the primary goal (4, 5). For this matter, the use of five-aminolevulinic acid (5-ALA) (6) in fluorescence-guided surgery (FGS) has significantly improved HGG therapy (7).

Since the first observational study of 5-ALA fluorescence-guided surgery for the resection of HGG (8) followed by a promising prospective study (9), a prospective phase III trial was able to show that its use significantly improved progression-free survival with 41% in the 5-ALA and 21.1% in the white light group at 6 months (10). Furthermore, 5-ALA has also been **used** for the visualization of other entities like meningiomas and/or metastases, although more appealing and strong evidence for its use in these lesions is needed (11, 12).

Nonetheless, some disadvantages exist. Blue light is used for excitation of the protoporphyrin IX (PpIX) fluorescence and is strongly absorbed by blood cells, as it has a strong attenuation to hemoglobin (6, 13). Even a single layer of erythrocytes can absorb the light and make the underlying PpIX fluorescence invisible to the surgeon. In the literature, this fact is described as a minor problem during surgery when continuous rinsing is applied (6). However, at final inspection of the surgical cavity, coagulated blood can easily masque malignant tumor tissue (6, 14). Hence, the surgeon needs to repeatedly switch between white and blue light in order to both detect bleeding sites and remove the tumor. Furthermore, if blood is lying above the tissue the view under blue light becomes dark and it gets more difficult to remove any blood or remaining tumor. If one does so, there is a high risk of destroying more vulnerable blood vessels existing in malignant tumor tissue when trying to treat bleeding with coagulation. Also, it is easier and more intuitive for the surgeon to pay attention to the total visual field under white light as opposed to blue light. Apart from that, there is an ergonomic problem when it is essential to repeatedly switch between white and blue light especially if heavy bleeding occurs. Hence, one could argue that it would be necessary to combine the advantages of both white and blue light in the setting of brain surgery with 5-ALA in order to diminish their disadvantages.

To do so, the multispectral fluorescence microscopy (MFL) was incorporated into a commercially available microscope (ARveo, Leica Microsystems, Wetzlar, Germany). We have already investigated its use for the combination of indocyanine green (ICG) fluorescence and white light images in the therapy of vascular anomalies of the brain in both an animal model, and on a human cadaver model as well as in clinical use for vascular and tumor applications (15–17). Such a microscope also allows simultaneous visualization of PpIX fluorescence and white light. After merging, this combined image is then delivered via the ocular and shows both the fluorescent properties of the inspected field and the anatomical details as visualized under white light (13, 17).

To the best of our knowledge, no other observational study has used MFL for the intraoperative visualization of brain tumors accumulated with PpIX fluorescence. As the MFL mode uses the information obtained from the blue light image, we hypothesized that the intraoperative assessment of the fluorescence quality and contrasts in the MFL mode would match the fluorescence under blue light. Thus, outcome parameters of this study were the matching information of both fluorescent pictures and intraoperative usability of the MFL mode when assessing and resecting a brain tumor. With this study, we want to get first insights into the use of MFL as a possible tool that could diminish the technological and ergonomical disadvantages of the visualization of PpIX fluorescence under blue light for fluorescence-guided surgery of brain tumors.

## Materials and Methods

The present study was approved by the ethics committee of the University of Witten/Herdecke, Germany (Nr: 35/2017). All patients participating in this prospective observational study gave written informed consent and were recruited in the Department of Neurosurgery of the Cologne Medical Center, Cologne, Germany. The follow-up was performed in the usual manner for every patient that is treated because of a brain tumor in our clinic.

Inclusion criteria for this study were:

- Primary or secondary brain tumor on an MRI, regardless of WHO grading
- no contraindication for the administration of 5-ALA
- histopathological confirmation of a brain tumor (primary and secondary)
- Written informed consent
- Age 18–75 years although we included also elderly patients if the Karnofsky index higher than 80%.

Exclusion criteria were:

- Severe comorbid diseases that could negatively influence the participation in this study
- Therapeutically uncontrollable arterial hypertension (i.e., systolic pressure above 200 mmHg and diastolic pressure above 120 mmHg)
- Lung diseases that are related to complications during narcosis and anesthesia (such as cystic fibrosis, alpha-1-antitrypsin-deficiency, sarcoidosis, allergic alveolitis, tuberculosis, etc.)
- Patient that received an attenuated vaccine 14 days or the influenza vaccination 7 days prior to their participation in this study
- All comorbid diseases that could influence the participation in this study.

In total, we examined 25 patients (11 women and 14 men) that were operated over the course of 22 months (Table 1) between 2018 and 2019. A total of 26 lesions were extirpated and assessed with the novel technique (one patient was operated on two different regions and had two different craniotomies in the same setting). The average age at operation was 62.29 years. The underlying diagnoses that we summarized as HGG were glioblastoma (*n* = 6), anaplastic astrocytoma (*n* = 4), and anaplastic oligodendroglioma (*n* = 1). Apart from that, we investigated the off-label use of 5-ALA in metastases from bronchial carcinoma (*n* = 5), metastasis from colon carcinoma (*n* = 3), metastases from breast carcinoma (*n* = 1), meningioma (*n* = 2), ependymoma (WHO grade II, *n* = 1), acoustic neurinoma (*n* = 1), metastasis from a myxoid liposarcoma (*n* = 1), and metastasis from tongue carcinoma (*n* = 1) (Table 1).

**Table 1:** List of patients operated on with the additional use of the MFL-technique.

**Patient-Nr**.	**Gender**	**Age at operation**	**Tumor entity**	**Tumor location**	**Primary tumor or mutation status**	**Fluorescence quality**	**Postoperative new neurological deficit**	**MRI- contrast enhanced resection**
1	F	71	Metastasis	Left parietal	Adeno-ca lung	Capsule intensive, content barely	None	Complete
2	F	59	Metastasis	Right temporal	Sigma-ca GI tract	Very intensive	None	Complete
			Metastasis	Right parietal	Sigma-ca GI tract	Very intensive	None	Complete
3	F	83	Metastasis	Right cerebellar	Adeno-ca lung	Intensive but inhomogeneous	None	Complete
4	M	63	Metastasis	Right parietal	Large cell ca lung	Very intensive	None	Complete
5	M	41	Anaplastic Astrocytoma	Right parietal	IDH-mutate, MGMT +	Very intensive	None	Minimal rest near postcentral
6	F	72	GBM cystic	Right temporal	IDH-mutate, MGMT +	Cyst very intensive, content barely	None	Minimal ependymal rest around the ventricle wall
7	F	75	Meningioma WHO I	Left frontal		Very intensive	None	Complete
8	M	50	Anaplastic Oligodendroglioma	Right frontal	MGMT +	Very intensive	None	Complete
9	M	56	Metastasis	Right frontal	Adeno-ca lung	Very intensive	None	Complete
10	M	53	GBM	Right temporal	IDH-wild type, MGMT +	Very intensive	None	Complete
11	M	77	Metastasis	Right frontal	Sigma-ca GI tract	Very intensive	None	Complete
12	F	77	Meningioma WHO I	Right parietal		Very intensive	None	Complete
13	F	51	Acoustic Neuroma	Right cerebelo-pontine angle		Intensive but inhomogeneous	Hypakusis	Complete
14	M	73	GBM	Right frontal	MGMT −	Very intensive	None	Complete
15	M	62	GBM	Right parieto-occipital	IDH-wild type, MGMT −	Very intensive	None	Minimal rest in the calcarine sulcus
16	M	44	Anaplastic Astrocytoma	Left trigonum, thalamus, midbrain	IDH-wild type, MGMT −	Very intensive	None	Incomplete resection due to midbrain thalamus infiltration
17	M	63	Metastasis	4th ventricle	Adeno-ca lung	Very intensive	Diziness	Complete
18	M	73	GBM	right frontal	IDH-wild type, MGMT −	Very intensive	None	No MRI postop
19	M	50	Ependymoma WHO II	4th ventricle		Very intensive	None	Complete
20	M	51	Metastasis	Spinal	Myxoid liposarcoma	Intensive but inhomogeneous	None	Complete
21	F	78	Metastasis	Left temporal	Squamous tongue ca	Very intensive	None	Complete
22	F	32	Anaplastic Astrocytoma	Left frontal precentral	IDH-mutate, MGMT +	Very intensive	None	Complete
23	M	65	Anaplastic Astrocytoma	Left frontal precentral	IDH-mutate, MGMT +	Very intensive	None awake surgery	Complete
24	F	76	GBM	Right frontal precentral	IDH-wild type, MGMT +	Very intensive	Hemiparesis arm 4/5	Minimal rest precentral, intraoperative monitoring +++
25	F	63	Metastasis	Right frontal	Breast-ca	Very intensive	None	Complete

Ca = Carcinoma, GBM = Glioblastoma, GI = gastrointestinal, IDH = isocitrate dehydrogenase, M = male, MGMT = O^6^-methylguanine DNA methyltransferase, F = female.

*In patient number 2, two different craniotomies had to be performed as the tumor was metastasized into both the temporal and parietal lobe. Patient 10 in this table is described in case vignette #1, patient 23 in case vignette #2 and patient 21 in case vignette #3*.

All patients received an oral dosage of 20 mg 5-ALA per kilogram body weight at least 3 h before the surgical procedure. Post-operatively, all patients were treated on the operative intensive care unit of our clinic before being moved to the general ward and kept in an area with low light exposition due to the phototoxic properties of 5-ALA.

### Technical Considerations

The MFL system, mounted on a conventional microscope (ARveo, Leica Microsystems, Wetzlar, Germany), consists out of a fluorescence excitation filter covering the absorption spectrum of PpIX in broad range around 405 nm, but blocking the PpIX emission peak at 635 nm to acquire the red fluorescence signal with a separate fluorescence camera. The PpIX fluorescence signal is processed into a pseudo color image with high contrast to the operative field (e.g., green) and transparently overlaid to the stereoscopic, optical view in the eyepieces (Leica Captiview). In addition, the pseudo color fluorescence image can be recorded and observed as embedded information in the white light image on the monitor. The microscope control allowed switching directly from white to blue light or MFL mode or from blue light to MFL mode and vice versa for surgical workflow support and comparison purposes.

### Operative Procedure

Following the craniotomy, we continued with a sharp surgical incision of the dura mater. Once the brain parenchyma was visualized macroscopically, we started to continue the procedure under the operation microscope (ARveo, Leica microsystems, Wetzlar Germany). If according to the MRI and intraoperative neuronavigation (Stealth Station, Medtronic, USA) the lesion was on the surface of the brain we continued under blue light in order to assess PpIX fluorescence and coherently visualize and identify the tumor. If the tumor was exposed, we changed to MFL-mode and observed the operation field and evaluated if the fused MFL image matched the different properties of the white light and blue light images. In tumors lying more deeply in the brain parenchyma we first operated under white light until the lesion was exposed. Then, we continued in the same fashion as described before.

When we suspected slight bleeding inside the surgical field during resection under blue light we changed to white light and assessed bleeding. After that, we turned to the MFL-mode in order to evaluate both the fluorescent properties of the tumor tissue and the bleeding. To achieve hemostasis, we switched back to white light. We evaluated the surgical field in the MFL-mode only in the case of slight bleedings that masked the fluorescence under blue light in order to not jeopardize the patient. Larger bleedings were immediately treated under white light. We repeated this procedure in the cases where small bleedings occurred while meticulously resecting the lesion under blue light.

During final inspection of the cavity under blue light we changed to white light to do a final assessment of coagulated blood or small bleeding that both could conceal tumor tissue. Then we switched to the MFL-mode to assess if the bleeding sites were matching the ones identified under white light. After that, we turned to blue light again. If any previously masked lesions were then identified we resected them accordingly until no fluorescent tissue was visible. Following the final inspection, we continued with sufficient hemostasis and closure of the surgical wound under white light. At the end of the operation, we assessed the resected tumor *ex vivo* under blue light and then changed to MFL-mode for the assessment of its fluorescent properties and if the characteristics of both images match. After each operation, the operating surgeon (CC) assessed the raw data (green fluorescence in a black picture, similar to what one sees when using the blue light in the classical way), which is calculated based upon the blue light fluorescence data. This was then compared to the MFL picture regarding matching fluorescence areas and shades.

## Results

As the software was installed in a surgical microscope the operative use of the MFL technique was easy to implement in the clinical setting. To correlate the overlay of fluorescence between the traditional blue light mode and the MFL mode the surgeon did an intraoperative assessment. Before switching to the MFL mode, the PpIX fluorescence under blue light was assessed for correspondence to the MFL picture.

In all 26 surgeries, we were able to correlate the pink fluorescence under blue light with the “green” fluorescence in the MFL-mode. The intensity of the green indicated fluorescence was always similar to the intensity of pink seen under blue light. In contrast to the conventional blue mode, under the MFL mode bleeding did not cause any problem as the sources of bleeding were immediately identifiable and were treated accordingly. Thus, periodic switching back to the white light was not necessary during the resection under the MFL fluorescence mode, which facilitated resection and surgical convenience especially in the situations that were ergonomically or surgically challenging (such as when larger bleedings occurred or at the final inspection).

Postoperatively, we reviewed the raw fluorescence signal video recordings and MFL video. Again, the perceived intensities and locations of pseudo colored green fluorescence overlay in the MFL mode corresponded perfectly with the single channel fluorescence recordings in all 26 surgeries. There was no instance when the natural color of the surgical field of view, reflections from the brain tissue or cerebrospinal fluid would impede fluorescence overlay visualization or create a false positive pseudocolored green fluorescence overlay.

In three cases, PpIX fluorescence using the MFL technique was intensive but inhomogeneous (see Table 1). In one metastasis from a bronchial carcinoma, the capsule fluorescence was very intense while its content lacked fluorescent intensity. In one GBM case, the cyst capsule displayed a strong fluorescent while the content only slightly fluoresced. In the remaining 20/25, the fluorescence quality was very intensive, while different shades of fluorescence were observed depending on the tumor site, i.e., center vs. periphery. The quality of the fluorescence signal displayed in the MFL mode was subjectively similar to the fluorescence under blue light using the traditional filter. Hence the different fluorescent shades representing different tumor areas were displayed correctly (Supplementary Video 1). This is the case because the data used to generate the MFL picture is taken from the visual information under blue light while simultaneously combining this information with the white light picture.

In 3/25 cases a new neurological deficit was found after the procedure. One patient was operated during awake surgery. In one patient, the intraoperative monitoring was positive and thus the surgeon decided to leave a minimal tumor residual in the precentral region. This patient then presented with a new hemiparesis of the left arm (muscle strength 4/5).

Regarding the degree of resection, postoperative MRI confirmed complete resection in 20/26 lesions. The extent of resection was determined using postoperative T1 weighted MRI scans with contrast. Those were compared with the preoperative MRI scans for residual contrast enhancement. In five lesions, a minimal residual was still present on the MRI. For one patient no postoperative imaging study was available.

### Case 1: Glioblastoma (Figure 1)

A 53-year-old male reported to our clinic with persisting headaches, amnesia, concentration deficits, confusion, and coordination deficits on the left side. This was accompanied by a tendency to fall to the left and insecure gait. The initial CT scan obtained showed a lesion in the right temporal-parietal region with significant surrounding edema. The cranial MRI scan revealed a lesion highly suspicious for GBM. We, therefore, scheduled the patient for the resection of the lesion. Prior to the intervention the patient received 20 mg per kilogram bodyweight of 5-ALA. Microsurgical resection of the tumor was performed with the modified microscope and concurring sonographic navigation and neuronavigation. The use of the MFL mode showed a coherent fusion of both white light and blue light images of the highly fluorescent tumor tissue and allowed for easy assessment of any bleedings occurring throughout resection. A tissue sample of the resected tumor was sent to the affiliated department of neuropathology and revealed a GBM. After the operation at the final neurological examination the patient presented himself with no neurological deficits and was totally mobile. Postoperative MRI showed a complete resection of the tumor.

**Figure 1:**
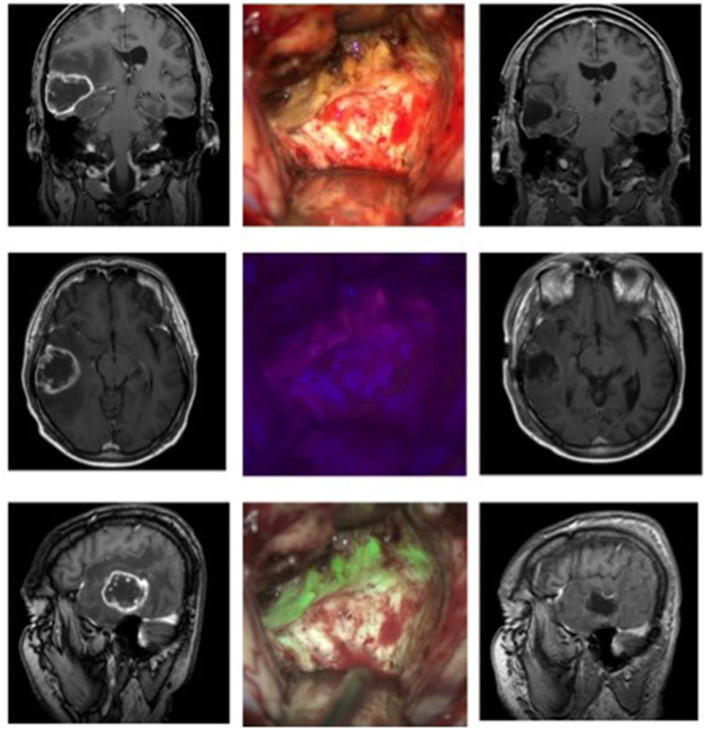
Glioblastoma in a 53-year-old patient. The left column shows the MRI images (T1 weighted with contrast, from top to bottom: coronary, axial, sagittal) before the operation, the right column the postoperative MRI. In the middle column, the top picture shows the surgical field under white light, below it under blue light, and at the bottom the fusion of both images in the MFL-mode.

Supplementary File shows a short segment of the operation of this patient.

### Case 2: Anaplastic Astrocytoma WHO Grade III (Figure 2)

A 65-year-old male initially presented himself with temporary hemiparesis of the right side accompanied by nausea. An initial MRI showed a hyperintense lesion on T2 in the left medial cingulate gyrus. We scheduled the patient for a biopsy of the lesion, which revealed an anaplastic astrocytoma (IDH-mutation, MGMT+, WHO grade III). After being discharged to his home prior to the elective surgery the patient presented himself through our emergency room with focal epileptic seizures of the right leg, namely the thigh region, causing local pain and cramps. We then decided to resect the lesion as soon as possible. Prior to the intervention the patient received 20 mg per kilogram bodyweight of 5-ALA. We planned neurophysiological monitoring of the right arm and leg during the operation to secure neurological function of the nearby supplementary motoric cortex. Microsurgical resection of the tumor was performed following by neurophysiological monitoring and neuronavigation. After the lesion was identified in the depth of the longitudinal fissure the use of the MFL mode showed a coherent fusion of both the white light and blue light images of the highly fluorescent tumor tissue and was able to assess bleedings occurring throughout resection. The resection was finished without any complications. After the operation, neurological examination revealed a light right leg hemiparesis, which was in remission on the day of discharge. Postoperative MRI showed a complete resection of the tumor.

**Figure 2:**
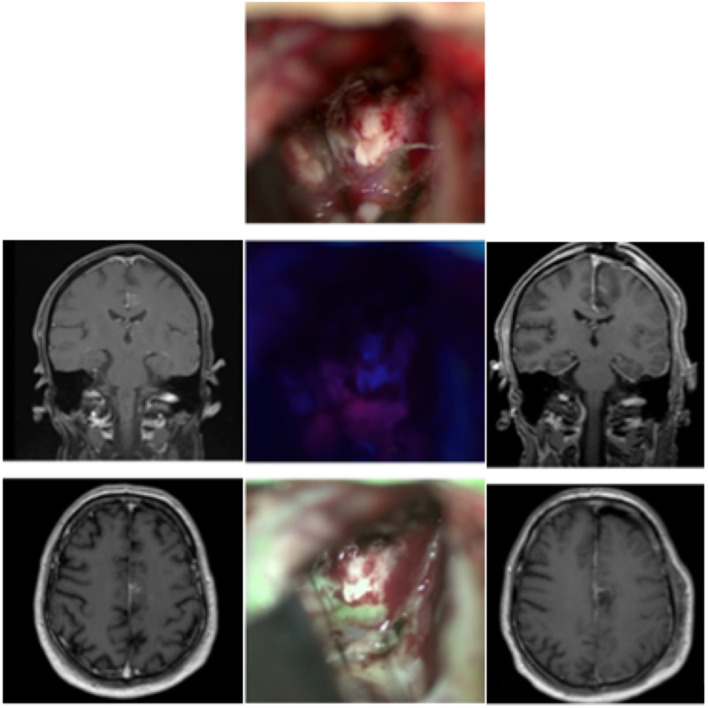
Anaplastic astrocytoma in a 65-year-old patient. The left side shows the preoperative MRI (contrast-enhanced), while the right column shows the early 48 h-postoperative MRI. In the middle column the operative cavity shows the field under white light, below it under blue light, and at the bottom the fusion of both images in the MFL-mode.

### Case 3: Metastases From a Squamous Carcinoma of the Tongue (Figure 3)

A 78-year-old female suffering from a known squamous carcinoma of the tongue came via the emergency room of our clinic because of a decline in her general condition accompanied by confusion and dizziness for the previous 3 weeks. The conducted MRI showed a necrotic and cystic lesion in the left temporal region with a midline shift. We, therefore, planned resection of the symptomatic temporal lesion. A CT scan obtained for disease staging showed several pulmonary lesions and potential lymph node metastases in the mediastinum as well as a lesion in the left gluteal region and inguinal lymph nodes, all highly suspicious of metastases. The patient received 5-ALA. The use of the MFL mode showed a coherent fusion of both the white light and blue light images of the highly fluorescent metastatic lesion tissue and was able to assess bleedings occurring throughout resection. The resection was finished without any complications and at the end no fluorescence was detected. The histological diagnosis revealed metastatic cells of the squamous carcinoma of the tongue. Postoperative MRI showed a complete resection of the tumor.

**Figure 3:**
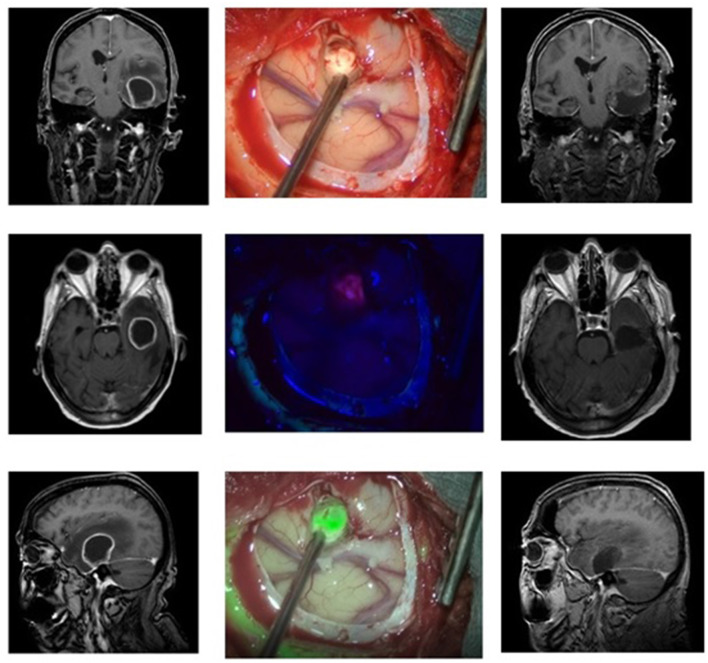
Metastasis from a squamous tongue carcinoma in a 78 old patient. Preoperative (left side) and postoperative MRIs (right side) showing the lesion and the total resection zone, respectively. In the middle column, the operative cavity is seen. The top picture shows the exposed tumor under white light, below it under blue light with pink nuances, and at the bottom the fusion of both images in the MFL-mode (“green” is the tumor in pseudo-color mode).

## Discussion

Even though the use of 5-ALA in neurosurgical patients was reported for the first time almost 20 years ago, fluorescence-guided surgery is, on the one hand, standard of care for brain tumor surgery, while on the other hand, still a developing field, especially in the improvement of fluorescence visibility from the technical point of view. This is highlighted by the fact that 5-ALA has only recently been approved by the FDA (18) and that it is currently used mainly for resection of HGG. Although 5-ALA has improved the outcomes of oncological neurosurgery, it comes with several technical disadvantages that need to be overcome.

To the best of our knowledge, the present observational study is the first investigation that describes a promising new technique that could help to improve PpIX fluorescence-guided tumor surgery while being easy to implement in the operation room. As hypothesized, intraoperative blue light images matched the MFL images while the new visualization technique did not violate operation ergonomics. On the contrary, as we have shown in Supplementary Video 1, drawbacks of the traditional blue filter such as blood covering the surgical field were more easily controllable. As we have illustrated in great detail through our small sample and the three illustrative cases, the MFL technique is able to visualize and combine both the picture generated under blue light as well as the white light picture in real-time. Thereby it enables the surgeon to assess the tumor and the surrounding neuroanatomy under white light. Through this, the ergonomic problem of switching between the different light modes and the need to continuously rinse the operation in order to prevent slight bleeding from covering the operation site could be solved. Most importantly, the ability to assess bleeding sites while also operating tumor tissue enables the surgeon to identify regions in real-time that could masque tumor tissue when blood coagulation has happened. Apart from that, learning to resect tumors in this augmented reality setting could prove to be more intuitive as opposed to the traditional way under blue light.

Furthermore, previously to this study we made the observation that this mode is able to sufficiently differentiate between the brain's physiological color and the artificial color chosen (15). This led us to believe that in the clinical setting of tumor surgery it would be easier to identify and resect tumor tissue in this augmented reality setting as opposed to operation under blue light were brain tissue is pretty much dark. We also made the same observation in a previous implementation of the MFL-mode for surgery of vascular malformations as well as tumors with indocyanine green (16).

In a recent paper, it was shown that the intensity of the PpIX fluorescence is related to the light source used, which, according to the authors, could have implications for surgery (19). This is further highlighted by the fact that various surgical microscopes can exhibit “considerably different illumination optical power levels at identical system settings” (20). According to Belykh et al. (20), this is the case because of several factors that are related to [1] the excitation light, as microscopes have variations in excitation light power which are related to several factors, [2] the detection of the emitted light, depending on optical parameters such as the distance between object and objective, sensitivity of the camera chip/eye, etc., and [3] factors related to the tissue itself, such as blood or light scatter, to name a few (21). Being aware of the effect of the light source upon the fluorescence properties of PpIX, we have tried to minimize this effect by undertaking several measures. All operations were performed by the same surgeon (CC), the microscope used was always identical, the angle between the light source and the surface of interest was always tried to be kept at 90° for the best illumination effect. Apart from that, the parameters that influence the fluorescence (namely illumination, magnification, and working distance) were tried to be kept as constant as possible. Only if operative challenges had to be met, the surgeon changed them accordingly to her needs.

Apart from the means used by the surgeon to cope with the problem of light intensity depending on the light source used, some technical points have to be mentioned and discussed. PpIX is excited with blue light of a narrow spectral range around 405 nm. Illuminated with such light the healthy tissue can be observed as a blue image. With increasing tumor cell density, the red fluorescence marking is added to the reflected blue light and colorizes the observed tissue detail from pinkish blue to strong pink (tissue with low up to strong PpIX fluorescence).

This color shift is generally independent of the excitation intensity, allowing the interpretation for surgical resection as long as the color visibility is given, but with further decrease of excitation intensity the visibility of the observed color signal becomes too dark and recognition of the faint fluorescence in the resection area is more or less impossible.

Comparing the PpIX MFL-white light fluorescence illumination used in the present study to the blue light fluorescence, the excitation of white light fluorescence is broad and comprising the wavelengths shorter than 400 nm up to 700 nm, excluding the PpIX emission peak around 635 nm for the detection of the PpIX fluorescence.

Because the detected fluorescence intensity is related to the light source excitation level in the absorption range of PpIX and the optical parameters, the MFL system allows the control of the related effects as much as possible to provide correct fluorescence intensity and visibility for the combined digital anatomical white light and fluorescence image on the monitor and for the fluorescence image overlay in the eyepieces.

In this observational study, we also report tumors other than HGG that showed PpIX fluorescence even though we applied 5-ALA in an “off-label” setting. Such lesions were different metastatic processes, meningiomas, acoustic neurinoma as well as ependymoma. Although in the literature, mainly HGGs are regarded as tumors that express the highly fluorescent PpIX and hence have fluorescent properties under blue light evidence exists that other brain lesions can also be visualized with 5-ALA, such as brain metastases (22). Of note, this seems to be only the case in approximately 50% of metastases while, interestingly, in one recent study, neither the primary site of the metastases nor their histologic subtype correlated with fluorescence behavior (22). Hence, we believe that with more knowledge about the different metastases and their 5-ALA metabolism this drug will eventually become more important for the field of neurosurgical oncology. Therefore, s it is crucial to address well-evaluated and documented disadvantages of 5-ALA regarding operation ergonomics and bleeding complications. The described novel MFL technology is able to bring light back to the operating field during the 5-ALA guided surgery, similarly to what we have previously described for fluorescence guided surgery using other fluorophores like ICG even for vascular pathologies (15) or brain tumor treatment (16). For that matter, the presented real time augmented reality setting combined with a commercially available microscope could be a promising tool to improve surgical therapy.

Nonetheless, the present study bears a few limitations. Being an observational study, we were only able to get the first insight into the way the MFL-mode could be used in neurosurgery. All pictures obtained in the MFL-mode were matching the fluorescence pictures under blue light while, however, the fluorescence and its contrast to the surrounding brain tissue was subjectively perceived stronger in the MFL-mode as opposed to the blue light pictures. In other words, we did not include any objective data such as quantitative assessment of fluorescence intensities or histopathological analysis of the tumor zones and the areas that showed different fluorescence intensity. Hence, future studies would need to quantitatively compare blue light and white light pictures to the generated MFL-mode picture regarding factors such as fluorescence intensity, discrimination of different tissues in real-time, or/and simultaneously identification of anatomically relevant structures such as blood vessels, to name a few. Apart from that, it is on our next steps to compare the traditional way of operating with both blue and white light to the sole use of the MFL-mode toward clinical endpoints such as progression-free survival, neurological function, or degree of tumor resection.

## Conclusion

The MFL technique embedded on a classic surgical microscope opens the way for precise and clear visualization of brain tissue PpIX fluorescence in real-time under white light. It can be easily implemented in the resection of all brain lesions accumulating 5-ALA and producing PpIX. Drawbacks of classic PpIX fluorescence visualization techniques such as difficulty in identification of bleeding sources, operating in dark environment and the necessity to regularly switch off the fluorescence mode could be overcome with the presence of additional white light which allows for clear simultaneous visualization of the exposed brain in natural colors and PpIX fluorescence in green pseudocolor.

## Data Availability Statement

All datasets generated for this study are included in the article/Supplementary Material.

## Ethics Statement

The studies involving human participants were reviewed and approved by University of Witten/Herdecke, Germany (Nr: 35/2017). The patients/participants provided their written informed consent to participate in this study. Written informed consent was obtained from the individual(s) for the publication of any potentially identifiable images or data included in this article.

## Author Contributions

PP and PC drafted the manuscript. PC and AH designed the manuscript. All operations were carried out by PC and MN. All authors read and approved the final manuscript.

## Conflict of Interest

The authors declare that the research was conducted in the absence of any commercial or financial relationships that could be construed as a potential conflict of interest.

## Supplementary Material

Supplementary Video 1 Intraoperative video as recorded with the operative microscope. The video was recorded during resection of the glioblastoma described in the case vignette #1. The video shows in the beginning the classic blue fluorescence while later on the multispectral fluorescence (MFL) mode is visible. The fluorescent tissue appears in the pseudo-color green. In this video, the different fluorescent shades are clearly visible, which helps identifying different areas of the lesion. It also shows that any blood covering the surgical field is immediately visible and can then be removed without losing focus of the tissue of interest. Finally, coagulated blood can be identified during dissection and then be removed while immediately seeing any underlying fluorescent tissue.
